# Prolonged Culture of Aligned Skeletal Myotubes on Micromolded Gelatin Hydrogels

**DOI:** 10.1038/srep28855

**Published:** 2016-06-28

**Authors:** Archana Bettadapur, Gio C. Suh, Nicholas A. Geisse, Evelyn R. Wang, Clara Hua, Holly A. Huber, Alyssa A. Viscio, Joon Young Kim, Julie B. Strickland, Megan L. McCain

**Affiliations:** 1 Laboratory for Living Systems Engineering, Department of Biomedical Engineering, USC Viterbi School of Engineering, University of Southern California, Los Angeles, CA, 90089, USA; 2 Oxford Instruments Asylum Research, Santa Barbara, CA, 93117, USA; 3 Keck School of Medicine of USC, University of Southern California, Los Angeles, CA, 90033, USA; 4 Department of Stem Cell Biology and Regenerative Medicine, Keck School of Medicine of USC, University of Southern California, Los Angeles, CA, 90033, USA

## Abstract

*In vitro* models of skeletal muscle are critically needed to elucidate disease mechanisms, identify therapeutic targets, and test drugs pre-clinically. However, culturing skeletal muscle has been challenging due to myotube delamination from synthetic culture substrates approximately one week after initiating differentiation from myoblasts. In this study, we successfully maintained aligned skeletal myotubes differentiated from C2C12 mouse skeletal myoblasts for three weeks by utilizing micromolded (μmolded) gelatin hydrogels as culture substrates, which we thoroughly characterized using atomic force microscopy (AFM). Compared to polydimethylsiloxane (PDMS) microcontact printed (μprinted) with fibronectin (FN), cell adhesion on gelatin hydrogel constructs was significantly higher one week and three weeks after initiating differentiation. Delamination from FN-μprinted PDMS precluded robust detection of myotubes. Compared to a softer blend of PDMS μprinted with FN, myogenic index, myotube width, and myotube length on μmolded gelatin hydrogels was similar one week after initiating differentiation. However, three weeks after initiating differentiation, these parameters were significantly higher on μmolded gelatin hydrogels compared to FN-μprinted soft PDMS constructs. Similar results were observed on isotropic versions of each substrate, suggesting that these findings are independent of substrate patterning. Our platform enables novel studies into skeletal muscle development and disease and chronic drug testing *in vitro*.

Skeletal muscle accounts for 30–40% of human body mass and is essential for movement and survival^1^. In humans, skeletal muscle mass decreases approximately 8% with each decade of age^2^, contributing to decreased mobility and quality of life for elderly populations. Patients with inherited skeletal myopathies, such as Duchenne Muscular Dystrophy (DMD), suffer from accelerated muscle degeneration and severe loss of mobility^3^. DMD is prevalent in approximately 1 out of 3500 male births per year and currently has no cure^3^. Currently, animal models are routinely used to identify mechanisms of skeletal muscle degeneration and disease and as model systems for preclinical drug screening^4^,^5^,^6^,^7^. Although animal models are ideal for detecting organism-level responses, they are low-throughput and expensive. Furthermore, identifying direct cause-effect relationships is challenging in animal models due to the number of confounding factors. Thus, there is a need for engineered *in vitro* skeletal muscle constructs as platforms for modeling skeletal muscle diseases and screening drugs with higher throughput and lower cost.

To culture skeletal muscle *in vitro*, proliferative skeletal myoblasts have traditionally been seeded onto synthetic culture substrates, such as polystyrene^8^,^9^ or polydimethylsiloxane (PDMS)^10^,^11^,^12^, that are coated or micropatterned (μpatterned) with extracellular matrix protein to facilitate cell adhesion. Myoblasts are then cultured in high-serum growth media until they reach confluence, at which point the media is changed to low-serum differentiation media. Over the next several days, myoblasts fuse into multi-nucleated myotubes and express skeletal muscle markers, such as sarcomeric α-actinin. However, previous studies using matrix-coated synthetic substrates for myoblast culture and differentiation have reported delamination of skeletal myotubes after approximately one week^8^,^10^,^11^,^13^. Delamination prohibits the use of these engineered constructs for long-term studies of skeletal muscle development and disease, including drug testing on chronic time scales.

Previously, we developed micromolded (μmolded) gelatin hydrogels cross-linked with microbial transglutaminase (MTG) as substrates for engineering neonatal rat and human induced pluripotent stem cell (iPSC)-derived cardiac tissues^14^. Gelatin solutions form hydrogels at room temperature when the concentration is above approximately 2% w/v, but liquefy when warmed to 37 °C^15^. With the addition of MTG, gelatin is cross-linked into thermostable hydrogels that can be used for cell culture^16^,^17^. Importantly, MTG is non-toxic, unlike many chemical cross-linkers, such as glutaraldehyde^16^,^17^. By altering the concentration of gelatin and MTG, the elastic modulus of gelatin hydrogels can be tuned to mimic the elasticity of native cardiac or skeletal muscle^14^. Furthermore, gelatin is naturally adhesive to most cells because it is derived from collagen, which is one of the extracellular matrix components of both cardiac^18^ and skeletal muscle^19^. Gelatin hydrogels can also be μmolded with PDMS stamps to induce tissue alignment via topographical cues, which is especially important for mimicking the architecture of striated muscle tissue^14^,^20^. Due to some or all of these beneficial features, engineered cardiac tissues remain viable and contractile on μmolded gelatin hydrogels for four weeks. This is a significant improvement over PDMS microcontact printed (μprinted) with fibronectin (FN), which maintain functional cardiac tissues for only one week^14^.

In this study, we modified our previous protocol for fabricating μmolded gelatin hydrogels cross-linked with MTG as cell culture substrates. We used atomic force microscopy (AFM) to characterize surface topography as well as changes in the surface elastic modulus due to incubation in culture-like conditions. We then tested if μmolded gelatin hydrogels would extend the culture lifetime of engineered skeletal myotubes differentiated from mouse C2C12 skeletal myoblasts compared to FN-μprinted PDMS and a softer blend of FN-μprinted PDMS. We chose the softer blend of PDMS because it is similar in composition to FN-μprinted PDMS, but similar in elasticity to gelatin hydrogels. After one week and three weeks in culture, we quantified overall cell density, the myogenic index, and the width and length of myotubes. On FN-μprinted PDMS, cells typically delaminated prior to the one week time-point, precluding myotube formation. Conversely, after one week in culture, myotubes were detected on both FN-μprinted soft PDMS and μmolded gelatin hydrogels with comparable myogenic index, width, and length. However, after three weeks in culture, the myogenic index and myotube width and length were significantly higher on μmolded gelatin hydrogels compared to FN-μprinted soft PDMS. We also cultured and differentiated myoblasts on isotropic versions of each substrate and observed similar trends, indicating that our results were independent of substrate topography or alignment. Together, our results suggest that gelatin hydrogels significantly improve the long-term adhesion of skeletal myotubes compared to the synthetic substrates we tested in this study. Our platform enables a variety of studies into mechanisms of skeletal muscle development, degeneration, and disease, and can be used to establish skeletal muscle drug screening platforms that are stable in culture for extended periods of time.

## Results

### Fabrication and Characterization of μmolded Gelatin Hydrogels

**Figure 1:**
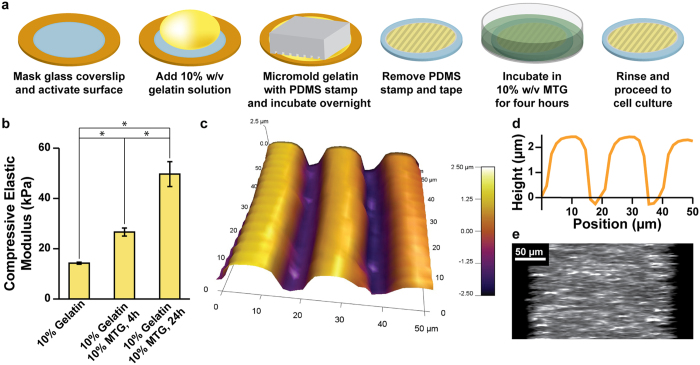
Fabrication and characterization of μmolded gelatin hydrogels. (**a**) Glass coverslips (blue) were masked with low-adhesive tape (orange), laser-engraved to selectively expose the center region, and chemically activated to facilitate gelatin adhesion. Gelatin solution (yellow) was dropped on the coverslip and molded with a PDMS stamp (gray). After overnight incubation, the stamp and tape were removed. The construct was then incubated in MTG solution (green) for four hours, rinsed, sterilized, and seeded with cells. (**b**) 10% w/v gelatin solutions were cast in Petri dishes and solidified into hydrogels after overnight incubation at room temperature. The bulk compressive elastic modulus of hydrogel cylinders was measured immediately (no MTG) or after incubation in the 10% w/v MTG solution for four or 24 hours at room temperature (mean ± s.e.m., n = 4 for each sample, *indicates statistically significant difference, ANOVA followed by Tukey’s test for multiple comparisons, p < 0.05). Details of statistical analysis are located in Supplementary Table S1. (**c**) AFM scan of the surface of a μmolded gelatin hydrogel construct. Color bar indicates height. (**d**) Values for the height of the cross-section of a μmolded gelatin hydrogel construct, illustrating the dimensions of the μmolded features. (**e**) Cross-section of a μmolded gelatin hydrogel construct doped with fluorescent beads, illustrating the overall height of the hydrogel construct.

Previously, we fabricated μmolded gelatin hydrogels cross-linked with MTG as substrates for engineering aligned cardiac tissues that remained adherent and functional for over three weeks^14^. The goal for the present study was to determine if similar substrates could extend the culture lifetime of engineered skeletal myotubes. Towards this goal, we developed a new fabrication procedure (shown schematically in Fig. 1a) to overcome technical challenges related to gelatin hydrogel cross-linking. Gelatin powder consists of partially hydrolyzed collagen and is readily soluble in 65 °C water at concentrations up to 20% w/v. Upon cooling to room temperature, solutions greater than 2% w/v solidify into hydrogels. However, pure gelatin hydrogels are not thermostable and will liquefy if warmed to 37 °C^21^, such as in a cell culture incubator. Previously, we and others have added MTG directly to gelatin solutions at 65 °C, which cross-links gelatin into a thermostable hydrogel that can be used as a cell culture substrate^14^,^16^,^17^. However, the addition of MTG directly to a gelatin solution limits the time for downstream fabrication steps because gelatin-MTG solutions cross-link relatively quickly compared to solutions of pure gelatin. Thus, we asked if it was possible to first solidify pure gelatin hydrogels at room temperature and then immerse the pre-formed hydrogels into solutions of MTG for cross-linking, also at room temperature. To test this, we cast 10% w/v gelatin solutions warmed to 65 °C into Petri dishes, allowed the solutions to solidify into a hydrogel due to overnight incubation at room temperature, and used biopsy punches to remove cylindrical samples. We then immersed select cylinders in 10% w/v MTG solutions for four hours or overnight at room temperature. When incubated at 37 °C, cylinders consisting of pure gelatin hydrogel liquefied, whereas the cylinders immersed in MTG for either four hours or overnight did not (data not shown), suggestive of cross-linking.

To determine if MTG immersion affected the mechanical properties of the hydrogels, we measured the bulk elastic modulus in compression of gelatin cylinders subjected to immersion of MTG for defined periods of time. As shown in Fig. 1b (statistical analysis in Supplementary Table S1), the average bulk elastic modulus of pure gelatin cylinders was 14.2 kPa ± 0.4 kPa (s.e.m., *n* = 4). Four-hour cross-linking with MTG increased the bulk elastic modulus to 26.6 kPa ± 1.6 kPa (s.e.m., *n*  =  4). Overnight cross-linking with MTG further increased the bulk elastic modulus to 49.7 kPa ± 5.0 kPa (s.e.m., *n* = 4). These increases in elastic modulus are further evidence that MTG was cross-linking the gelatin hydrogel. Additionally, this data indicates that the duration of MTG cross-linking affects the elastic modulus of the resulting hydrogel, which is a relatively simple way to tune its elasticity. Thus, we successfully cross-linked pure gelatin hydrogels that were pre-formed at room temperature by immersion into MTG solutions, which affords more time for downstream fabrication procedures, such as μmolding, as described below.

Our next goal was to fabricate μmolded gelatin hydrogels as cell culture substrates, using our new cross-linking technique and adapting our previously published protocol^14^ (Fig. 1a). First, we used low-adhesive tape and a laser engraver to mask the edges of glass coverslips, which were chemically activated to promote gelatin adhesion. Next, we pipetted pure gelatin solution (10% w/v, 65 °C) onto the exposed portions of coverslips. We chose 10% w/v gelatin solution because hydrogels with lower concentrations were generally not sufficiently robust to survive the μmolding process. Similar to our previous fabrication method, we μmolded the surface by placing a 10×10 PDMS stamp with 2 μm-deep features on top of the gelatin drop. The PDMS stamp was gently pressed until it reached the taped edges to displace excess gelatin solution and form a relatively uniform slab of hydrogel on the coverslip. Our rationale for choosing PDMS stamps with 10 × 10 dimensions is that 10 μm is the approximate width of a cell and thus 10 × 10 should sufficiently align myoblasts, similar to our previous experience with cardiac myocytes^14^. We chose PDMS stamps with a relatively shallow depth of 2 μm so that the available surface area for cell adhesion would be relatively un-changed between μmolded and flat surfaces, thereby minimizing surface area as a potential variable when comparing μmolded constructs to flat constructs. After incubating PDMS stamps on gelatin solutions overnight at room temperature, we hydrated constructs, carefully removed the stamps, and immersed constructs in a solution of MTG (10% w/v) at room temperature for four hours. We chose four-hour incubation with MTG because the elastic modulus of native skeletal muscle has been reported within 10–50 kPa^19^,^22^,^23^. Thus, we selected a formulation with an elastic modulus similar to native skeletal muscle tissue, based on our previous measurements (Fig. 1b). Constructs were then rinsed in PBS and stored at 4 °C until use.

Next, we asked if features from the PDMS stamp robustly transferred to the surface of the gelatin hydrogel during the μmolding process. To answer this, we used AFM to generate a height map of the surface of a μmolded gelatin hydrogel. As shown in Fig. 1c,d, the μmolded features were approximately 10 μm wide with 10 μm spacing and a depth of approximately 2 μm. Thus, the features from the PDMS stamp were transferred relatively faithfully to the surface of the hydrogel. This data also indicates that the μmolded features on the surface of the hydrogel are likely not deep enough to support layering of cells within the depressed features.

To measure the overall thickness of μmolded gelatin hydrogels, we embedded fluorescent beads within a construct and collected confocal z-stacks. As shown in Fig. 1e, the entire gelatin hydrogel was thicker than 200 μm, which is mostly dictated by the thickness of the tape used to mask the glass coverslip during fabrication (Fig. 1a). Thus, the hydrogel has sufficient thickness to minimize the effects of the underlying rigid glass coverslip.

### Degradation of Cross-Linked Gelatin Hydrogels in Culture-Like Conditions

**Figure 2:**
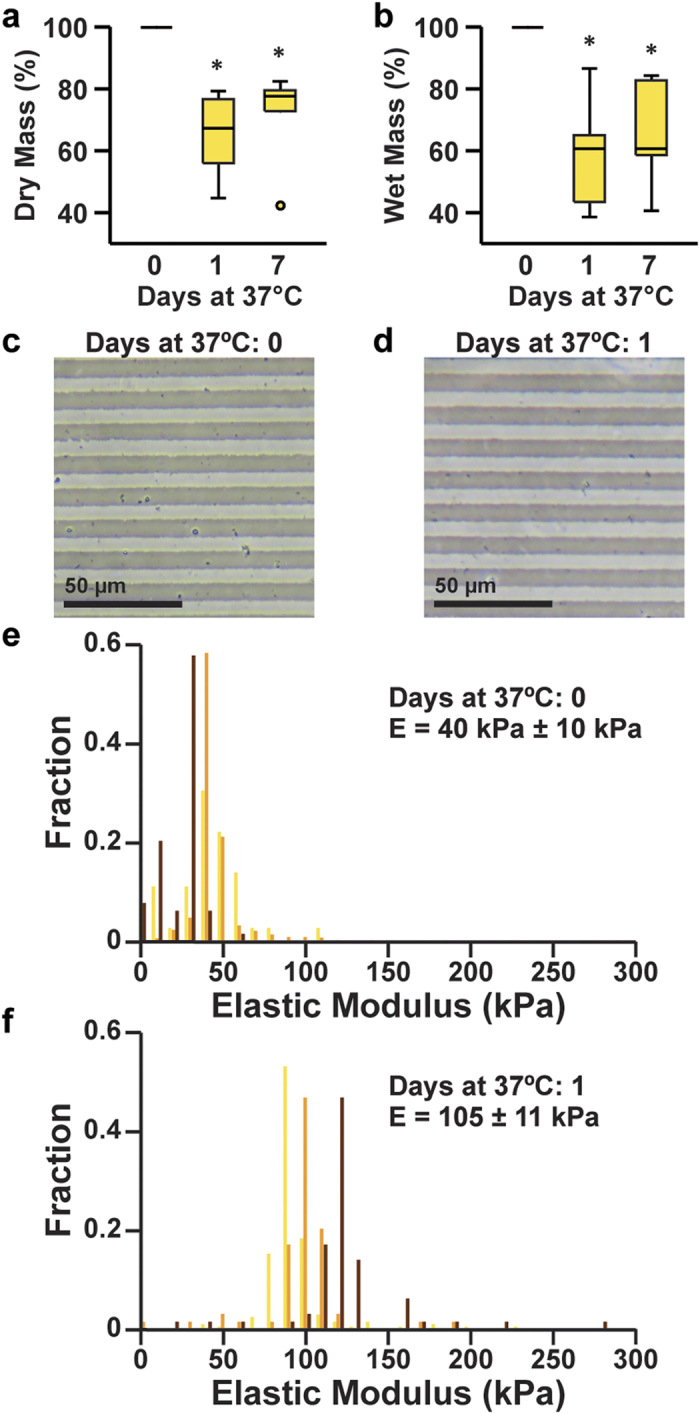
Degradation of μmolded gelatin hydrogels in culture-like conditions. Measurements of the relative dry mass (**a**) and relative wet mass (**b**) of μmolded gelatin hydrogels after incubation for the indicated time in PBS at 37 °C (*n* = 7 for zero days and one day, *n* = 6 for seven days, *indicates statistically significant difference compared to zero days, Kruskal-Wallis test followed by Tukey’s test for multiple comparisons, p < 0.05). Details of statistical analysis are located in Supplementary Tables S2 and S3. Features μmolded onto gelatin hydrogels were present both before (**c**) and after (**d**) overnight incubation in PBS at 37 °C. Histograms of elastic moduli values collected from μmolded gelatin hydrogels incubated for zero days (**e**) or one day (**f**) in PBS at 37 °C. Each color represents values recorded from an independent construct (*n* = 3 for 0 days and 1 day). Average elastic modulus values ± s.e.m. are indicated on the plots.

We next asked if cross-linked gelatin hydrogel constructs would degrade in culture-like conditions. To test this, we fabricated μmolded gelatin hydrogel constructs adhered to glass coverslips, using the methods described above. We then incubated these constructs in PBS at 37 °C and measured changes in wet and dry mass after one day and one week. As shown in Fig. 2a,b (statistical analysis in Supplementary Tables S2 and S3), the dry mass was 58% ± 6.9% (s.e.m., *n* = 7) of the initial dry mass and the wet mass was 66% ± 5.3% (s.e.m., *n* = 7) of the initial wet mass after one day at 37 °C. This is likely due to an immediate loss of un-cross-linked gelatin from constructs upon warming to 37 °C. However, there was no significant difference in the wet or dry mass between constructs incubated for one day and one week at 37 °C (p > 0.05, Kruskal-Wallis test), indicating that the mass was relatively stable after the first day in culture-like conditions. To characterize the thermostability of the μmolded features on the surface of the hydrogel, we imaged μmolded hydrogel constructs before and after overnight incubation in PBS at 37 °C overnight. The μmolded features were intact on the surface of the hydrogel both before (Fig. 2c) and after (Fig. 2d) incubation, indicating that μmolded features were preserved despite the loss of mass.

Due to the loss of mass that we observed, we next asked if the elasticity of the cross-linked gelatin hydrogels would also change due to incubation in culture-like conditions. To obtain elasticity measurements that would more precisely reflect those experienced by cells, we measured the elasticity of the surfaces of μmolded gelatin hydrogels using AFM. Because we observed mass changes only after the first day in culture-like conditions, we chose to measure pristine μmolded gelatin hydrogel constructs and constructs incubated overnight at 37 °C. For each construct, we collected multiple measurements of elastic modulus to minimize sampling error. As shown in Fig. 2e,f, we plotted the elastic modulus values from each independent construct as a histogram and calculated the average elastic modulus for each construct. The overall average elastic modulus of pristine μmolded gelatin hydrogel constructs was 40 kPa ± 10 kPa (s.e.m., n = 3). It is important to note that this value is higher than the values we recorded using bulk compression testing of hydrogel cylinders (Fig. 1b), which could be due to differences in measurement techniques and/or actual differences in the bulk versus surface elastic moduli. After overnight incubation at 37 °C, the elastic modulus increased significantly to 105 kPa ± 11 kPa (s.e.m., *n* = 3, p = 0.0495, Kruskal-Wallis test). Thus, the elastic modulus of μmolded gelatin hydrogels increases after one day in culture-like conditions. Although this increase in elastic modulus renders the constructs slightly stiffer than physiological values, they are still more similar to the elasticity of native skeletal muscle when compared to many synthetic materials traditionally used as cell culture substrates, such as glass, polystyrene, or PDMS.

### Adhesion of C2C12 Skeletal Myoblasts on PDMS, Soft PDMS, and Gelatin Hydrogels

Previous studies have reported that skeletal myotubes delaminate from synthetic culture substrates, such as PDMS, coated with matrix proteins after approximately one week^8^,^10^,^11^. As described above, our goal was to determine if μmolded gelatin hydrogels could be utilized as substrates for prolonging the culture of engineered skeletal myotubes. We first tested the biocompatibility of the μmolded gelatin hydrogels cross-linked with MTG by seeding C2C12 mouse skeletal myoblasts on these constructs. After one day in culture, we quantified cell viability by staining live and dead cells (Supplementary Figure 1). We calculated cell viability as 96.6% ± 0.8% (s.e.m., *n* = 3), indicative of minimal cytotoxicity on μmolded gelatin hydrogels.

We next asked if μmolded gelatin hydrogels would improve cell adhesion compared to FN-μprinted PDMS. To generate FN-μprinted PDMS, we spin-coated PDMS onto glass coverslips and μprinted FN as 15 μm-wide lines separated by 2 μm-wide gaps. We chose this pattern because it was previously shown to be optimal for engineering confluent, aligned neonatal rat cardiac myocyte tissues^24^. Because FN-μprinted PDMS and μmolded gelatin hydrogels differ considerably in both elastic modulus and composition, we also μprinted FN on a softer blend of PDMS, as previous reports have suggested that reducing the elastic modulus can improve myotube adhesion^22^. We fabricated soft PDMS by mixing PDMS with Sylgard 527 silicone dielectric gel at a 1:20 mass ratio, similar to previously published protocols^25^. The elastic modulus of PDMS and soft PDMS in compression was measured as 2503 kPa ± 39.4 kPa (s.e.m., *n* = 4) and 27.3 kPa ± 1.2 kPa (s.e.m., *n* = 6), respectively. Thus, FN-μprinted soft PDMS constructs were more similar in elasticity to gelatin hydrogels, but more similar in composition to FN-μprinted PDMS constructs. To evaluate the effects of alignment and/or topography, we also generated isotropic versions of each substrate. For PDMS and soft PDMS, we fabricated isotropic constructs by coating surfaces uniformly with fibronectin solution. For gelatin hydrogels, we fabricated isotropic constructs by molding the gelatin hydrogel surface with a featureless PDMS stamp. Thus, in total, we fabricated six types of culture substrates: FN-μprinted PDMS, FN-isotropic PDMS, FN-μprinted soft PDMS, FN-isotropic soft PDMS, μmolded gelatin, and isotropic gelatin.

**Figure 3:**
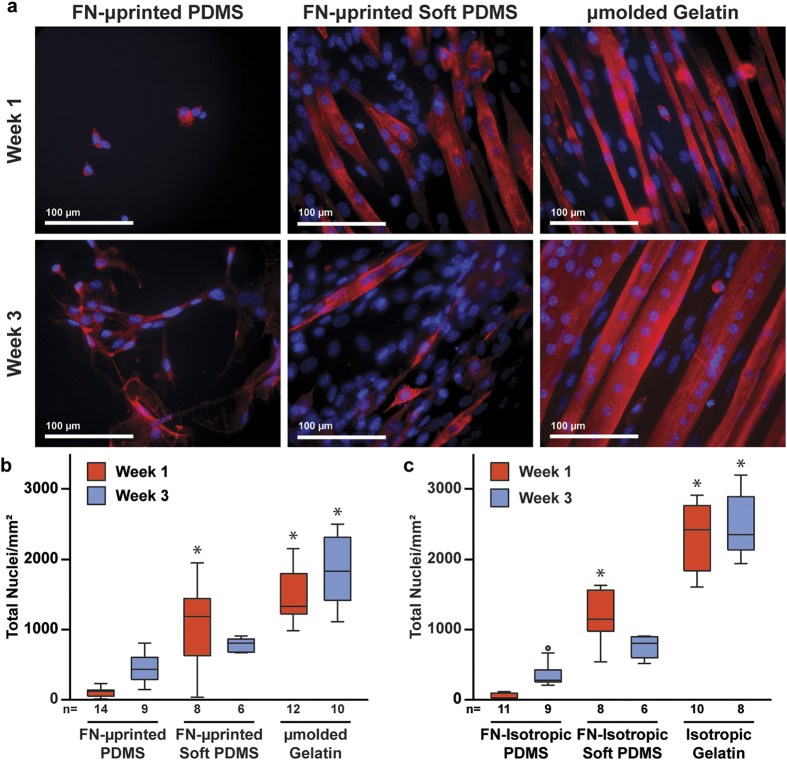
Cell adhesion on engineered constructs. (**a**) Representative images of C2C12 skeletal myoblasts seeded and differentiated into myotubes on FN-μprinted PDMS (first column), FN-μprinted soft PDMS (second column), and μmolded gelatin hydrogels (third column). Images were collected one week (first row) and three weeks (second row) after initiating differentiation of myoblasts into myotubes. Blue: nuclei, red: sarcomeric α-actinin. Total number of nuclei present on μpatterned (**b**) and isotropic (**c**) constructs (n is indicated below each bar, *indicates statistically significant difference compared to PDMS construct at same time point, Kruskal-Wallis test followed by Tukey’s test for multiple comparisons, p < 0.05). Details of statistical analysis are located in Supplementary Tables S4 and S5.

To identify differences in cell adhesion due to the culture substrate, we seeded C2C12 mouse skeletal myoblasts onto the six types of substrates. After myoblast cultures reached confluence (3–4 days), we differentiated the cells into myotubes and fixed and stained constructs for nuclei and sarcomeric α-actinin after an additional one or three weeks in culture, which are referred to as Week 1 and Week 3, respectively. For each immunostained construct, we collected five fluorescent images with a high-power objective (field of view: 281.6 μm × 237.6 μm) and counted the number of nuclei in each image without discriminating between myoblasts and myotubes. Qualitatively, cell delamination was consistently observed on FN-μprinted PDMS and thus constructs were sparsely covered with cells at Week 1 and Week 3 (Fig. 3a). Conversely, on μmolded gelatin hydrogels, cells remained adherent at every time point. The number of nuclei on FN-μprinted soft PDMS appeared most similar to μmolded gelatin hydrogels compared to FN-μprinted PDMS. Quantitatively, the number of cells on μmolded gelatin was significantly higher than FN-μprinted PDMS at both Week 1 and Week 3. No statistical differences were detected between FN-μprinted soft PDMS and μmolded gelatin at Week 1 or Week 3 (Fig. 3b, statistical analysis in Supplementary Table S4). The number of cells on FN-μprinted soft PDMS was significantly higher than FN-μprinted PDMS only at Week 1. Similar trends were observed on isotropic constructs (Fig. 3c, statistical analysis in Supplementary Table S5). Thus, μmolded gelatin hydrogels and isotropic gelatin hydrogels improve cell adhesion compared to FN-μprinted PDMS and FN-isotropic PDMS, respectively. However, no significant differences were observed in cell adhesion between μmolded gelatin hydrogels and FN-μprinted soft PDMS, or isotropic gelatin hydrogels and FN-isotropic soft PDMS.

### Myotube Morphology on Gelatin Hydrogel and Soft PDMS Constructs

**Figure 4:**
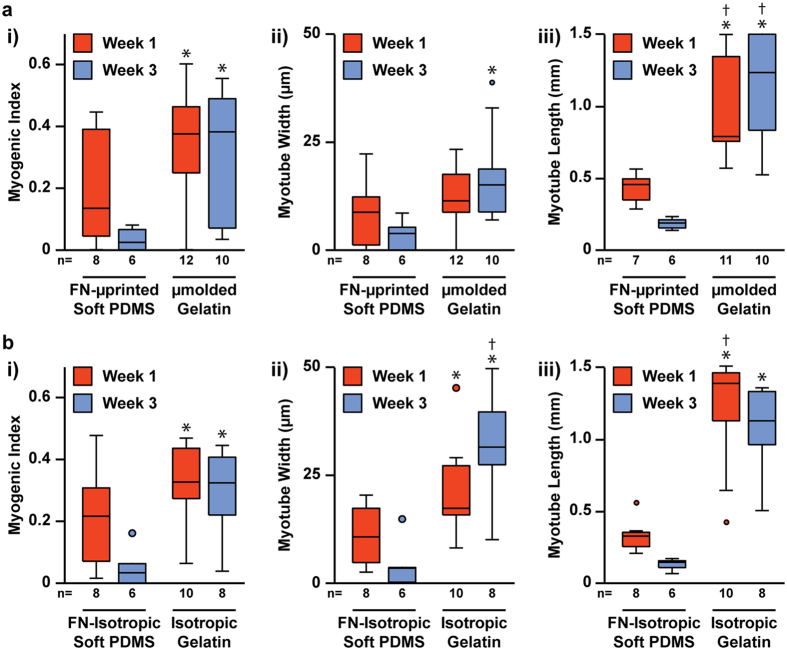
Quantification of myogenic index, myotube width, and myotube length on engineered constructs. Myogenic index (i), myotube width (ii), and myotube length (iii) for μpatterned (**a**) and isotropic (**b**) constructs one week and three weeks after initiating differentiation of myoblasts into myotubes (n is indicated below each bar, *indicates statistically significant difference compared to soft PDMS constructs at Week 1, ^†^indicates statistically significant difference compared to soft PDMS constructs at Week 3, Kruskal-Wallis test followed by Tukey’s test for multiple comparisons, p < 0.05). Detailed statistical analyses are located in Supplementary Tables S6–S11.

For the cell adhesion studies described above, we did not discriminate between nuclei in myoblasts versus differentiated myotubes. Thus, we next quantified if gelatin hydrogels improve the adhesion and morphology of myotubes specifically. We performed myotube analysis only for gelatin hydrogels and soft PDMS constructs because delamination precluded significant myotube formation on PDMS constructs. To compare myotube formation, we first calculated the myogenic index, which is defined as the number of nuclei within sarcomeric α-actinin-positive myotubes divided by the total number of nuclei. As shown in Fig. 4a (statistical analysis in Supplementary Table S6), the myogenic index at Week 1 was not significantly different between FN-μprinted soft PDMS constructs and μmolded gelatin hydrogels. However, at Week 3, the myogenic index was significantly higher on μmolded gelatin hydrogels compared to FN-μprinted soft PDMS constructs. Similar results were found when comparing FN-isotropic soft PDMS to isotropic gelatin (Fig. 4b, statistical analysis in Supplementary Table S7), suggesting that improvements in myogenic index are independent of μmolding or μprinting. Thus, the myogenic index on μmolded gelatin hydrogels and isotropic gelatin hydrogels was significantly higher at Week 3 compared to FN-μprinted soft PDMS constructs and FN-isotropic soft PDMS constructs, respectively.

**Figure 5:**
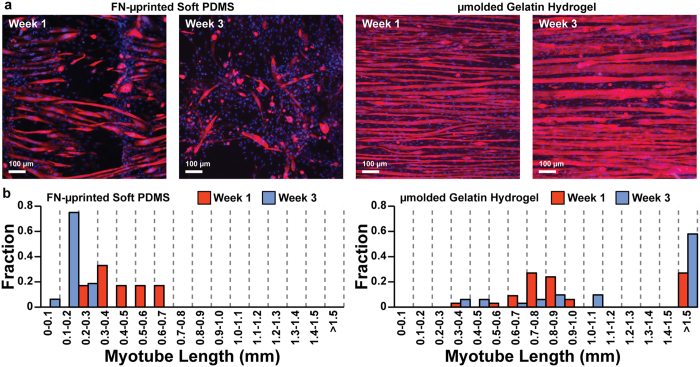
Myotube length on engineered constructs. (**a**) Representative images of C2C12 skeletal myoblasts cultured on FN-μprinted soft PDMS and μmolded gelatin hydrogels, stained one week (Week 1) and three weeks (Week 3) after initiating differentiation into myotubes. (**b**) Histograms of myotube lengths in FN-μprinted soft PDMS and μmolded gelatin hydrogels.

To further assess myotube formation, we measured and compared the average width and length of sarcomeric α-actinin-positive myotubes. At Week 1, there was no significant difference in myotube width between FN-μprinted soft PDMS and μmolded gelatin hydrogels (Fig. 4a, statistical analysis in Supplementary Table S8). However, at Week 3, myotubes were significantly wider on μmolded gelatin hydrogels compared to FN-μprinted soft PDMS. Similar results were observed on isotropic constructs (Fig. 4b, statistical analysis in Supplementary Table S9). To calculate myotube length, we collected three fluorescent images per construct with a low-power objective (field of view: 1.7 mm × 1.4 mm) to increase the size of the field of view. However, despite increasing the field of view, myotubes routinely extended beyond the image, especially on gelatin hydrogels (Fig. 5a). To capture this graphically, we plotted the average myotube length in each image (3 images per construct) as a histogram, with the last bin representing myotubes that were longer than the field of view (Fig. 5b). For the purposes of statistical analysis, we recorded myotube length for those extending beyond the field of view as 1.5 mm, which is likely an underestimate. However, because we performed statistical analysis on ranks, we expect that this would not significantly alter our conclusions. As shown in Fig. 4a (statistical analysis in Supplementary Table S10), myotubes were significantly longer on μmolded gelatin hydrogels compared to FN-μprinted soft PDMS constructs at both Week 1 and Week 3. Similarly, myotubes were significantly longer on isotropic gelatin hydrogels compared to FN-isotropic soft PDMS constructs at both Week 1 and Week 3 (Fig. 4b, statistical analysis in Supplementary Table S11). Together, these data indicate that the long-term adhesion and morphology of myotubes was significantly improved on μmolded gelatin hydrogels and isotropic gelatin hydrogels compared to FN-μprinted soft PDMS and FN-isotropic soft PDMS, respectively.

**Figure 6:**
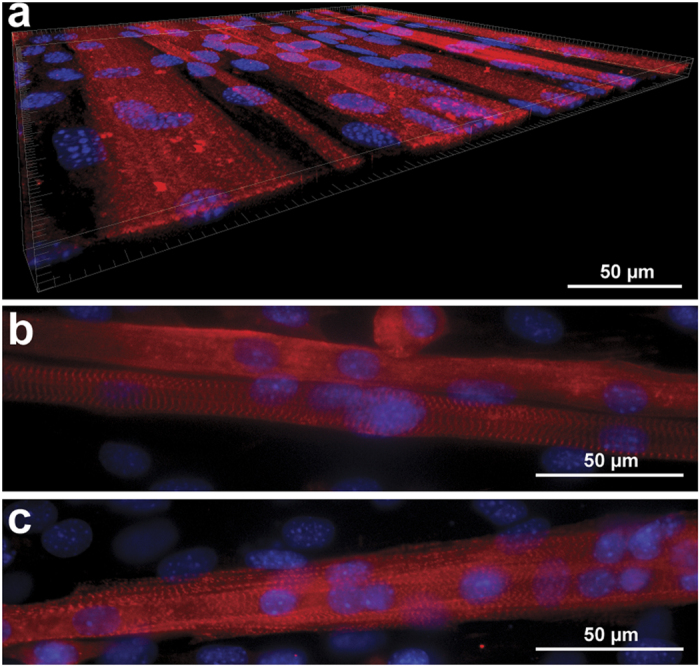
Tissue thickness and sarcomere formation in myotubes. (**a**) 3-D volume of skeletal myotubes cultured on μmolded gelatin hydrogels, illustrating that the tissue is a relatively flat monolayer. Myotubes with visible sarcomeres on μmolded gelatin hydrogels one week (**a**) and three weeks (**b**) after differentiation. Blue: nuclei, red: sarcomeric α-actinin.

As shown in Fig. 1c,d, the features on μmolded gelatin hydrogel had a depth of approximately 2 μm. To assess how the μmolded features altered the topography of the resulting tissue, we collected a confocal *z*-stack of myotubes cultured on μmolded gelatin hydrogels, deconvolved the image, and created a 3-D volume. As shown in Fig. 6a, the tissue was a relatively flat monolayer, likely because the μmolded features in the hydrogel were relatively shallow. Thus, μmolding the surface of the hydrogels did not introduce deep features that could potentially alter the number of cell layers in the resulting tissue.

Mature myotubes display striated sarcomeres when stained for sarcomeric α-actinin. However, in most of the myotubes we observed on our engineered constructs, sarcomeric α-actinin staining was diffuse. This pattern of staining is indicative of myotube immaturity and is consistent with previous C2C12 *in vitro* studies^26^. However, we did observe striated sarcomeric structures in select myotubes on μmolded gelatin hydrogels at both Week 1 (Fig. 6b) and Week 3 (Fig. 6c). Thus, with further optimization and/or longer time in culture, myotube maturation and sarcomerogenesis can likely be improved.

### Preserved Alignment of Skeletal Myotubes on μmolded Gelatin Hydrogels

**Figure 7:**
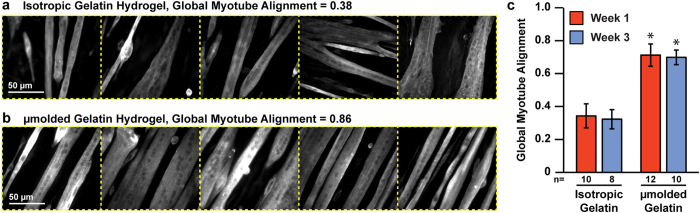
Global myotube alignment on isotropic and μmolded gelatin hydrogels. (**a,b**) For each coverslip, five images of sarcomeric α-actinin (white) were captured at multiple locations and assembled into a five-panel montage. Global myotube alignment was calculated as the orientational order parameter of vectors assigned to sarcomeric α-actinin intensities within each montage. Representative isotropic (**a**) and μmolded (**b**) montages with low and high global myotube alignment, respectively. Dotted yellow lines were added to illustrate boundaries between individual images. (**c**) Global myotube alignment for isotropic and μmolded gelatin hydrogels at each time point (mean ± s.e.m, *n* is indicated below each bar, *indicates statistically significant difference compared to isotropic gelatin constructs at same time point, ANOVA followed by Tukey’s test for multiple comparisons, p < 0.05). Detailed statistical analyses are located in Supplementary Table S12.

As described above, we μmolded the surface of our gelatin hydrogels to promote myotube alignment, with the goal of mimicking the striated architecture of native skeletal muscle. To determine if μmolding would induce and sustain the formation of aligned myotubes, we assembled the five high-resolution images (field of view: 281.6 μm × 237.6 μm) collected from a given construct into a single montage image (Fig. 7a,b) and calculated the orientational order parameter of each montage. We refer to this value as “global myotube alignment” because it captures the alignment of multiple locations on the same coverslip relative to each other. As shown in Fig. 7c (statistical analysis in Supplementary Table S12), global myotube alignment was significantly higher on μmolded gelatin hydrogels compared to isotropic gelatin hydrogels at both Week 1 and Week 3. Importantly, myotube alignment was not significantly different between Week 1 and Week 3 on molded gelatin hydrogels, indicating that myotube alignment was stable over the three week culture period.

## Discussion

Robust and sustainable *in vitro* models of skeletal muscle are critically needed to identify mechanisms of skeletal muscle development, degeneration, and disease in a controlled setting, and to serve as testbeds for pre-clinical drug screening. However, cultured skeletal myotubes routinely delaminate from matrix-coated synthetic culture substrates after approximately one week^8^,^10^,^11^,^13^. In this study, we successfully maintained aligned skeletal myotubes *in vitro* for three weeks by utilizing μmolded gelatin hydrogels as culture substrates. This platform enables a wide-range of studies related to skeletal muscle that were previously challenging, if not impossible, due to myotube delamination.

In our study, we compared the adhesion and structure of myotubes differentiated from C2C12 mouse skeletal myoblasts on six types of substrates: (1) FN-μprinted PDMS; (2) FN-isotropic PDMS; (3) FN-μprinted soft PDMS; (4) FN-isotropic soft PDMS; (5) μmolded gelatin hydrogels; and (6) isotropic gelatin hydrogels. PDMS and soft PDMS constructs consist of a synthetic silicone elastomer coated or μprinted with a layer of fibronectin^25^,^27^. Thus, these substrates differ primarily in elastic modulus. On FN-isotropic and FN-μprinted PDMS substrates, myoblasts initially adhered to the substrate, but consistently delaminated several days after fusing into myotubes. On FN-isotropic and FN-μprinted soft PDMS substrates, delamination was reduced and differentiated myotubes were detectable after one and three weeks in culture. These results suggest that decreasing the elastic modulus of the substrate reduces myotube delamination, similar to previous studies^22^. This effect was independent of cell alignment, as both FN-isotropic and FN-μprinted substrates produced similar results.

Gelatin is a natural biomaterial derived from collagen and therefore is inherently adhesive to cells^15^. In this study, we fabricated gelatin hydrogels with elastic moduli similar in magnitude to soft PDMS, especially when compared to PDMS. After three weeks in differentiation media, myogenic index, myotube width, and myotube length were each significantly higher on μmolded gelatin hydrogels and isotropic gelatin hydrogels compared to FN-μprinted soft PDMS and FN-isotropic soft PDMS, respectively. Because these trends were independent of μpatterning, we can conclude that differences in substrate topography and any subsequent increases in surface area for cell adhesion are not a dominant factor for the improvements in myotube formation and adhesion that we observed. One potential explanation for the benefit of gelatin hydrogels is that myoblasts and myotubes have a greater supply of matrix protein provided by the substrate itself. Thus, as cells remodel and/or degrade matrix protein on the surface, additional cell adhesion sites throughout the bulk of the hydrogel become available. Conversely, on FN-μprinted soft PDMS, once the cells remodel and/or degrade the single FN layer on the surface, the cells are exposed to bare PDMS. Cell adhesion to PDMS is low due to a lack of integrin adhesion sites and the hydrophobicity of PDMS^28^. However, further studies are needed to better control for other differences between FN-μprinted soft PDMS and μmolded gelatin hydrogels to fully understand the underlying mechanisms of our findings.

One important difference between FN-μprinted soft PDMS and μmolded gelatin hydrogels that was not explored in this study is that these substrates differ in the specific type of matrix protein available for cell adhesion. Gelatin and fibronectin recruit different integrin receptors, which can affect cell adhesion as well as cell signaling^29^,^30^. Gelatin consists of denatured collagen, which is the primary extracellular matrix protein in native skeletal muscle^19^, which could contribute to the improvements in myotube adhesion we observed. However, in native skeletal muscle, muscle fibers connect to collagen primarily through the glycoprotein laminin, which is the dominant component of the basement membrane^19^. In agreement with this finding, studies have shown that both mouse C2C12 and human skeletal myotube formation is enhanced on laminin-μprinted surfaces compared to surfaces μprinted with fibronectin, Type I collagen, or Type IV collagen^13^. Similarly, myoblast adhesion to Collagen I films was shown to be enhanced by coating the films with laminin^31^. These studies together suggest that laminin is a strong cue for myotube formation and adhesion, which was lacking from our constructs. Studies have also shown that myoblasts proliferate and differentiate on solubilized matrices isolated from decellularized muscle tissue^9^, which potentially provide the ideal mixture of ECM molecules for skeletal muscle adhesion, differentiation, and maturation. Beyond ECM composition, additional parameters related to the ECM that we did not explore in this study include the width, spacing, and depth of μprinted and/or μmolded features. Thus, further studies are needed to better understand how the composition and structure of the ECM together can be further optimized to promote myotube adhesion and maturation.

Another limitation of our study is that we focused exclusively on morphological parameters. However, for skeletal muscle especially, morphological parameters are relatively reliable indicators of tissue development and maturation^11^ and are commonly reported in the literature. Importantly, we routinely measured myotubes longer than 1.5 mm on μmolded gelatin hydrogels. For comparison, prior studies of C2C12 myoblasts report maximum myotube lengths below 500 μm^8^,^32^,^33^, with few studies reporting myotube lengths greater than 1 mm^13^,^34^. Thus, we have shown a substantial increase in myotube length compared to past approaches. We anticipate that these structural differences will translate into functional differences, which can be measured in future studies by adapting the muscular thin film assay^14^,^35^,^36^,^37^ to skeletal myotubes^11^.

Most of the myotubes that we observed displayed diffuse sarcomeric α-actinin staining instead of striated sarcomeric structures, which is consistent with other C2C12 *in vitro* studies^26^. However, we did observe sarcomeres in select myotubes, suggesting that myotubes are capable of forming mature sarcomeres on μmolded gelatin hydrogels. To further promote sarcomere maturation, the culture conditions could be improved by incorporating more features that mimic the native muscle environment, including biophysical cues. For example, myotube formation and maturation has been shown to improve when myoblasts are stimulated by either electrical pulses^38^,^39^ or optogenetic activation^26^. Both static^40^ and cyclic^32^ mechanical stretch have also been shown to increase myotube formation. Furthermore, myotube formation *in vitro* has been shown to be sensitive to a variety of signaling molecules and growth factors^41^, including microRNAs^42^ and cytokines^43^. The addition of supporting cell populations, such as neurons^44^, fibroblasts, or endothelial cells, will likely also promote myotube formation and maturation. Importantly, our platform is amenable to the addition of a variety of parameters for future investigation, with the long-term goal of engineering biomimetic skeletal muscle tissue constructs. Optimizing the extracellular matrix is only one essential component of this process.

Recently, 3-D models of skeletal muscle bundles have been established *in vitro* by allowing a mixture of myoblasts and matrix protein to self-assemble within PDMS molds^45^. These 3-D muscle bundles are likely more biomimetic than the 2-D myotube cultures described here. However, 3-D models require significantly more cells than our 2-D platform. 3-D tissues are also more challenging to image and are less amenable to high-throughput drug screening because they are relatively complex to assemble. Thus, it is still important to develop 2-D platforms, such as the one described in our study, as models of skeletal muscle that require fewer cells and are easier to fabricate and image compared to 3-D platforms.

For the experiments described here, we used C2C12 mouse skeletal myoblasts because these cells are well-characterized and widely used for *in vitro* studies. However, we anticipate that μmolded gelatin hydrogels will similarly prolong the culture lifetime of human skeletal myotubes, which could be used for both human-relevant drug screening and disease modeling. To culture human skeletal myotubes, myoblasts could be sourced from patient muscle biopsies^45^ or differentiated from human iPSCS^46^. Furthermore, to model human genetic skeletal myopathies, such as DMD, iPSCs harboring mutations associated with genetic skeletal myopathies could be created with gene editing^47^, eliminating the need to collect biopsies directly from patients. Establishing these models of human skeletal myotubes on μmolded gelatin hydrogels would avoid differences between species that have limited the relevance of animal models. For example, most DMD studies to date have used a mouse model, known as the mdx mouse, which does not fully capture the phenotype seen in human patients^48^. For modeling skeletal muscle pathologies induced by soluble factors, such as the insulin resistance observed in Type 2 Diabetes^49^, skeletal myotubes could be cultured on μmolded gelatin hydrogels inside a fluidic device as an “Organ on a Chip” system. With the addition of fluidic components, soluble factors could be added in a defined manner. A “Skeletal Muscle on a Chip” platform could also be fluidically coupled to other “Organ on a Chip” systems to capture organ-organ interactions and systemic drug responses^50^,^51^. Thus, our platform can be extended to a variety of studies for modeling skeletal muscle diseases caused by genetic and/or environmental factors.

In conclusion, we have established that μmolded gelatin hydrogels maintain aligned skeletal myotubes in culture for three weeks. This platform enables a variety of long-term studies into skeletal muscle development and disease and chronic testing of drugs. Future studies will focus on utilizing μmolded gelatin hydrogels as substrates for culturing human skeletal myotubes with the goal of developing human-relevant models of diseases, such as DMD, and integrating functional metrics, such as contractility.

## Methods

### Bulk Compressive Elastic Modulus Measurements

To measure the bulk compressive elastic modulus of gelatin hydrogels, 10% w/v gelatin solutions warmed to 65 °C were poured into Petri dishes and stored overnight at room temperature to solidify into a hydrogel. Gelatin hydrogel cylinders of 8 mm diameter were removed from Petri dishes using a biopsy punch. Cylinders were then immersed in a 10% w/v solution of MTG for zero, four, or twenty-four hours. The elastic modulus of three cylinders per dish were measured using an Instron 5942 Mechanical Testing System and averaged. Each cylinder was compressed 35% of its initial height. The 0–10% range of compressive strain was utilized for elastic modulus calculations. The height and radius of each cylinder was measured and input for measurements. To measure the compressive elastic modulus of PDMS and soft PDMS, both substrates were cured in Petri dishes. Cylinders were removed and measured using the same protocol described for gelatin hydrogels. For each substrate type, at least three independent samples were fabricated and measured.

### PDMS Stamp Fabrication

Standard photolithography and soft lithography protocols were used to make wafers and PDMS stamps^52^. Briefly, wafer templates were fabricated by spin-coating SU-8 2002 negative photoresist (MicroChem) onto silicon wafers and exposing the coated wafer to UV light through a custom photomask using a mask aligner. The photomask consisted of either 15 μm wide lines separated by 2 μm wide gaps (15 × 2) or 10 μm wide lines separated by 10 μm wide gaps (10 × 10). Wafers were then immersed in developer solution to remove un-exposed photoresist and coated with trichloro (1H,1H,2H,2H-perfluorooctyl) silane. PDMS was created by mixing the elastomer base and curing agent of Sylgard 184 silicone elastomer kit (Dow Corning) in a 10:1 mass ratio. PDMS was then poured over a wafer template in a Petri dish, de-gassed, and cured at 65 °C for at least four hours. Cured PDMS was then carefully removed from the wafer and cut into square stamps measuring approximately 2 cm × 2 cm.

### Fabrication of Gelatin Hydrogel Constructs

Similar to previous protocols^14^, 25 mm round glass coverslips were covered with tape and a circle located 1–2 mm inside the edge of the coverslip was cut with an Epilog Mini 24 Laser Engraver (30 Watt). The center region of tape was removed such that tape only covered the edges of the coverslip. Coverslips were then activated using 100 mM NaOH, 0.5% (3-Aminopropyl) trimethoxysilane, and 0.5% glutaraldehyde, similar to previous protocols^53^. After activation, coverslips were dried in a 65 °C oven for 5–10 minutes and stored at room temperature.

10% w/v gelatin solution was prepared by dissolving 175 g Bloom Type A porcine gelatin in distilled, deionized water warmed to 65 °C. The gelatin solution was mixed and degassed in a centrifugal Thinky mixer using 30 seconds of mixing and 20 seconds of degassing. The 10% w/v gelatin solution was added dropwise onto activated coverslips. 10 × 10 PDMS stamps or flat PDMS stamps cast in a Petri dish were sonicated in 95% ethanol, dried, pressed firmly on top of the gelatin, and incubated overnight at room temperature. The taped edges supported the edges of the stamps such that the thickness of the gelatin hydrogels was uniform and consistent^14^. The stamps were then removed and the tape was carefully peeled off the coverslip. Gelatin coverslips were then transferred to a 6- or 12-well plate and incubated in a 10% w/v solution of microbial transglutaminase (MTG) (Ajinomoto) dissolved in distilled, deionized water for four hours at room temperature. Coverslips were then washed in PBS, stored at 4 °C, and treated in a UVO cleaner for one minute to sterilize constructs immediately prior to cell seeding. Hydrogels molded with 10 × 10 stamps are referred to as μmolded gelatin hydrogels. Hydrogels molded with flat PDMS stamps are referred to as isotropic gelatin hydrogels. To generate hydrogels with fluorescent beads to measure hydrogel thickness, gelatin solution was doped with 0.2 μm FluoSpheres yellow-green 505/515 beads (ThermoFisher Scientific) at a concentration of 1:1000.

### Fabrication of PDMS and Soft PDMS Constructs

Two types of PDMS with different elastic moduli were fabricated, which are referred to as PDMS and soft PDMS. PDMS was created as described above. Soft PDMS was created by first mixing the A and B components of Sylgard 527 silicone dielectric gel (Dow Corning) in a 1:1 mass ratio. Sylgard 527 was then mixed with PDMS in a 20:1 mass ratio to generate soft PDMS, similar to published protocols^25^. All mixing steps were done using a centrifugal Thinky mixer with two minutes of mixing and two minutes of degassing. PDMS and soft PDMS mixtures were then spin-coated onto 18 mm or 25 mm round glass coverslips using a Specialty Coating Systems G3P-8 spin coater, similar to published protocols^54^. PDMS-coated coverslips were incubated in 65 °C for at least four hours to cure and then stored at room temperature.

15 × 2 PDMS stamps were sonicated in 95% ethanol for 15 minutes and dried using compressed air in a laminar flow hood. Human FN diluted in distilled, de-ionized water (50 μg/ml) was pipetted onto each stamp surface and incubated for one hour at room temperature. Immediately before microcontact printing, PDMS- and soft PDMS-coated coverslips were treated for eight minutes in a UVO cleaner. Coated stamps were then dried with compressed air, pressed firmly onto UVO-treated coverslips, and immediately removed. This process created PDMS- and soft PDMS-coated coverslips microcontact printed with FN, which are referred to as FN-μprinted PDMS and FN-μprinted soft PDMS, respectively. Additional UVO-treated PDMS- and soft PDMS-coated coverslips were coated isotropically with FN by placing coverslips upside-down onto FN drops for fifteen minutes. This process created PDMS- and soft PDMS-coated coverslips isotropically coated with FN, which are referred to as FN-isotropic PDMS and FN-isotropic soft PDMS, respectively. All coverslips were transferred to a 6- or 12-well plate, rinsed in PBS, and stored at 4 °C until cell seeding.

### Degradation Studies

To measure changes in the mass of gelatin hydrogel constructs due to culture-like conditions, excess liquid was dried from freshly made μmolded gelatin hydrogel constructs using a delicate task wiper and the construct was weighed. The average mass of a glass coverslip was subtracted. These values were recorded as the initial wet masses. Constructs were then incubated overnight at room temperature until dry and weighed again. After subtracting the average glass coverslip mass, these values were recorded as the initial dry masses. Constructs were then placed in a 37 °C incubator in PBS. After one day or one week, samples were weighed wet, left to dry overnight, and then re-weighed, using the same procedures described above. The wet mass and dry mass of each construct at each time point was divided by the initial wet mass and dry mass, respectively, to quantify changes in mass due to incubation in culture-like conditions. For each time point, at least three independent samples were fabricated and measured.

### Atomic Force Microscopy

Atomic Force Microscopy (AFM) measurements were performed on an MFP-3D Infinity (Oxford Instruments Asylum Research, Santa Barbara, USA). Silicon nitride (TR400; Olympus, Tokyo, Japan) cantilevers were calibrated using the GetReal contact-free calibration method^55^ for both sensitivity and spring constant. The measured spring constants for each cantilever used were within the expected range provided by the manufacturer. Force curves were measured in PBS. In order to minimize the effects of hydrodynamic drag and viscous effects, the velocity of the measurement was chosen such that the indentation, retraction, and non-contact portions of the approach and withdraw traces were identical. The measured modulus of each gel did not change by lowering the measurement speed below the chosen velocity. Using the built-in modeling software of the AFM, the plasticity and adhesion of each curve was measured in order to exclude the applicability of non-Hertzian contact models. The Hertz/Sneddon model was then applied to all acquired data using a cone-shaped indenter based on the geometry of unmodified silicon nitride. For each construct, three independent samples were fabricated and measured. The cross-section was derived from the force height map, which represents the height of the AFM tip when the maximum force trigger is reached. The height map was planefitted and flattened to remove tilt due to sample mounting. Both modifications were limited to first-order planefit and flatten in order to preserve the validity of the measured height data.

### Mouse C2C12 Skeletal Myoblast Culture and Differentiation

Mouse C2C12 skeletal myoblasts (ATCC) were seeded onto substrates at a concentration of 100,000 cells/well for 6-well plates and 60,000 cells/well for 12-well plates. Myoblasts were seeded and cultured in growth media, which consisted of Dulbecco’s Modified Eagle Medium (DMEM) with high glucose supplemented with 10% fetal bovine serum and 1% penicillin-streptomycin. Once confluent, C2C12 cells were incubated in differentiation media, which consisted of DMEM with high glucose supplemented with 2% horse serum and 1% penicillin-streptomycin. Cells were maintained in an incubator at 37 °C with 5% CO_2_. Growth or differentiation media was replenished every two days.

### Cell Viability Measurements

Cell viability was quantified with the ReadyProbes Cell Viability Imaging Kit, Blue/Green (ThermoFisher Scientific) using manufacturer’s instructions. Briefly, C2C12 skeletal myoblasts were seeded on μmolded gelatin hydrogels in 6-well plates with 3 mL of media. One day after seeding, constructs were incubated with two drops of NucBlue Live reagent, which stains all nuclei, and two drops of NucGreen Dead reagent, which stains only dead cells. Five fields of view per construct were captured using a Nikon Ti inverted fluorescence microscope. The number of total and dead nuclei was manually counted and cell viability was calculated by dividing the number of live nuclei by the number of total nuclei.

### Immunostaining

Cultured cells were fixed with 4% paraformaldehyde, permeabilized with 0.05% Triton-X, and incubated with monoclonal mouse anti-sarcomeric α-actinin (Sigma, 1:200) primary antibody for one hour at room temperature. After rinsing with PBS, cells were incubated with an Alexa Fluor 546 goat anti-mouse secondary antibody (Life Technologies, 1:200), 4′,6-diamidino-2-phenylindole (DAPI, 1:200), and Alexa Fluor 488 Phalloidin (Life Technologies, 1:200) for one hour at room temperature. All samples were mounted with ProLong Gold Anti-Fade Mountant (Life Technologies).

### Imaging and Image Analysis

For each coverslip, five locations dispersed across each construct were imaged using a 60x oil objective on a Nikon Ti inverted fluorescence microscope. Images were captured using an Andor Zyla scientific CMOS camera. Four types of image analysis were implemented in ImageJ and/or MATLAB: total number of nuclei, myotube width, number of nuclei in myotubes, and global myotube alignment. Total number of nuclei was determined by using the Cell Counter plugin in ImageJ and counting all DAPI-positive nuclei shown in a single field of view taken at 60x magnification (277 μm × 234 μm). Myotubes were identified by positive sarcomeric α-actinin staining and myotube width was measured in ImageJ. In each image, up to five representative measurements of myotube width were collected, depending on the number of myotubes present in the image. The number of nuclei in myotubes was determined by counting the number of nuclei that co-localized with positive sarcomeric α-actinin staining. Myogenic index was then calculated by dividing the number of nuclei in myotubes by the total number of nuclei. To quantify global myotube alignment, the five images collected for each coverslip were assembled into a single montage image in ImageJ. The orientation angles of continuous pixel segments were calculated for each montage image using custom MATLAB code, as previously reported^35^,^56^. The orientational order parameter^57^,^58^ was then calculated for each montage as a metric of global myotube alignment. To measure myotube length, three locations dispersed across each construct were imaged using a 10x air objective on the same microscope and camera system described above. In each image, up to five representative measurements of myotube length were collected using ImageJ, depending on the number of myotubes present in the image. For all measurements, at least six constructs per condition were tested, which were collected from at least three independent experiments.

Confocal *z*-stacks were collected using a Nikon C2 point-scanning confocal microscope system. To generate the cross-section of fluorescent beads within the gelatin hydrogel, gelatin hydrogels doped with fluorescent beads were imaged with a 10x air objective. The *z*-stack was opened and processed in ImageJ to create a cross-section image. To generate the 3-D volume of myotubes, an immunostained tissue was imaged with a 60x oil objective. The image was deconvolved using Hyugens Professional (Scientific Volume Imaging) and subsequently opened and processed in Imaris (BitPlane).

### Statistical Analysis

All measurements were first tested for normality using the Lilliefors Test. Data that was normally distributed was analyzed using one-way ANOVA followed by Tukey’s test for multiple comparisons in MATLAB, α set to 0.05. Data that was not normally distributed was analyzed using the Kruskal-Wallis test followed by Tukey’s test for multiple comparisons in MATLAB, α set to 0.05. The statistical test for each comparison is noted in the text or Figure legend and additional statistical analyses are located in the Supplementary Tables.

## Additional Information

**How to cite this article**: Bettadapur, A. *et al*. Prolonged Culture of Aligned Skeletal Myotubes on Micromolded Gelatin Hydrogels. *Sci. Rep*. **6**, 28855; doi: 10.1038/srep28855 (2016).

## Supplementary Material

Supplementary Information
