# Supplementary material for: Prolonged Culture of Aligned Skeletal Myotubes on Micromolded Gelatin Hydrogels
Source: Sci Rep. 2016 Jun 28;6:28855. doi: 10.1038/srep28855 (PMC4924097; doi:10.1038/srep28855)
Supplement: Supplementary Information [file srep28855-s1.pdf]

Supplementary Information for:

Prolonged Culture of Aligned Skeletal Myotubes on Micromolded Gelatin Hydrogels

Archana Bettadapur[1,2], Gio C. Suh[1,2], Nicholas A. Geisse[3], Evelyn R. Wang[1,4], Clara Hua[1], Holly A. Huber[1], Alyssa A. Viscio[1], Joon Young Kim[1], Julie B. Strickland[1], Megan L. McCain*[1,5]

[1]Laboratory for Living Systems Engineering, Department of Biomedical Engineering, USC Viterbi School of Engineering, University of Southern California, Los Angeles, CA 90089

[2]These authors contributed equally to this work

[3]Oxford Instruments Asylum Research, Santa Barbara, CA 93117

[4]Keck School of Medicine of USC, University of Southern California, Los Angeles, CA 90033

[5]Department of Stem Cell Biology and Regenerative Medicine, Keck School of Medicine of USC, University of Southern California, Los Angeles, CA 90033

* Corresponding Author:

Megan L. McCain
USC Viterbi School of Engineering
1042 Downey Way, DRB 140
Los Angeles, CA 90089
Phone: 213-821-0791
Email: mlmccain@usc.edu

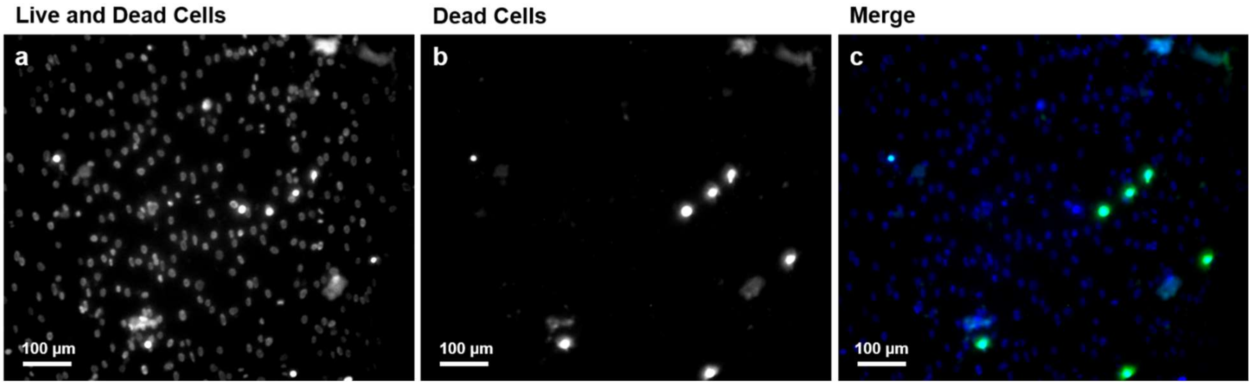

Supplementary Figure 1. Cell Viability on µmolded Gelatin Hydrogels. Representative image of C2C12 skeletal myoblasts cultured on µmolded gelatin hydrogels for one day and stained to quantify cell viability. Cells were stained to identify live and dead cells (a) and dead cells only (b). (c) Merged image shows live and dead cells in blue and dead cells in green.

Supplementary Table S1. Statistical analysis for compressive elastic moduli of gelatin hydrogels cross-linked with MTG. All data was normally distributed, as determined by the Lilliefors test. The *p*-value for one-way analysis of variance (ANOVA) was 1.7 x 10e-5. Multiple comparisons were performed using Tukey's test, with the *p*-values indicated in the table below.

Comparison	*p*-value
No MTG vs 10% MTG 4 hours	<0.03
10% MTG 4 hours vs 10% MTG 24 hours	<0.001
No MTG vs 10% MTG 24 hours	<0.0001

Supplementary Table S2. Statistical analysis for dry mass of µmolded gelatin hydrogel constructs. All data was not normally distributed, as determined by the Lilliefors test. The *p*-value for the Kruskal-Wallis test was 8.4 x 10e-4. Multiple comparisons were performed using Tukey's test, with the *p*-values indicated in the table below.

Comparison	*p*-value
0 day vs 1 day	<0.001
0 day vs 7 days	<0.03
1 day vs 7 days	NS

Supplementary Table S3. Statistical analysis for wet mass of µmolded gelatin hydrogel constructs. Data was not normally distributed, as determined by the Lilliefors test. The *p*-value for the Kruskal-Wallis test was 0.0011. Multiple comparisons were performed using Tukey's test, with the *p*-values indicated in the table below.

Comparison	*p*-value
0 day vs 1 day	<0.005
0 day vs 7 days	<0.01
1 day vs 7 days	NS

Supplementary Table S4. Statistical analysis for total nuclei in µpatterned tissues. All data was not normally distributed, as determined by the Lilliefors test. The *p*-value for the Kruskal-Wallis test was 1.1 x 10e-8. Multiple comparisons were performed using Tukey's test, with *p*-values for statistical differences indicated in the table below.

Comparison	*p*-value
Wk1 FN-µprinted PDMS vs Wk3 FN-µprinted PDMS	NS
Wk1 FN-µprinted PDMS vs Wk1 FN-µprinted soft PDMS	<0.02
Wk1 FN-µprinted PDMS vs Wk3 FN-µprinted soft PDMS	NS
Wk1 FN-µprinted PDMS vs Wk1 µmolded gelatin	<0.00001
Wk1 FN-µprinted PDMS vs Wk3 µmolded gelatin	<0.00001
Wk3 FN-µprinted PDMS vs Wk1 FN-µprinted soft PDMS	NS
Wk3 FN-µprinted PDMS vs Wk3 FN-µprinted soft PDMS	NS
Wk3 FN-µprinted PDMS vs Wk1 µmolded gelatin	<0.03
Wk3 FN-µprinted PDMS vs Wk3 µmolded gelatin	<0.01
Wk1 FN-µprinted soft PDMS vs Wk3 FN-µprinted soft PDMS	NS
Wk1 FN-µprinted soft PDMS vs Wk1 µmolded gelatin	NS
Wk1 FN-µprinted soft PDMS vs Wk3 µmolded gelatin	NS
Wk3 FN-µprinted soft PDMS vs Wk1 µmolded gelatin	NS
Wk3 FN-µprinted soft PDMS vs Wk3 µmolded gelatin	NS
Wk1 µmolded gelatin vs Wk3 µmolded gelatin	NS

Supplementary Table S5. Statistical analysis for total nuclei in isotropic tissues. All data was not normally distributed, as determined by the Lilliefors test. The *p*-value for the Kruskal-Wallis test was 5.9 x 10e-9. Multiple comparisons were performed using Tukey's test, with *p*-values for statistical differences indicated in the table below.

Comparison	*p*-value
Wk1 FN-isotropic PDMS vs Wk3 FN-isotropic PDMS	NS
Wk1 FN-isotropic PDMS vs Wk1 FN-isotropic soft PDMS	<0.02
Wk1 FN-isotropic PDMS vs Wk3 FN-isotropic soft PDMS	NS
Wk1 FN-isotropic PDMS vs Wk1 isotropic gelatin	<0.00001
Wk1 FN-isotropic PDMS vs Wk3 isotropic gelatin	<0.00001
Wk3 FN-isotropic PDMS vs Wk1 FN-isotropic soft PDMS	NS
Wk3 FN-isotropic PDMS vs Wk3 FN-isotropic soft PDMS	NS
Wk3 FN-isotropic PDMS vs Wk1 isotropic gelatin	<0.01
Wk3 FN-isotropic PDMS vs Wk3 isotropic gelatin	<0.01
Wk1 FN-isotropic soft PDMS vs Wk3 FN-isotropic soft PDMS	NS
Wk1 FN-isotropic soft PDMS vs Wk1 isotropic gelatin	NS
Wk1 FN-isotropic soft PDMS vs Wk3 isotropic gelatin	NS
Wk3 FN-isotropic soft PDMS vs Wk1 isotropic gelatin	NS
Wk3 FN-isotropic soft PDMS vs Wk3 isotropic gelatin	NS
Wk1 isotropic gelatin vs Wk3 isotropic gelatin	NS

Supplementary Table S6. Statistical analysis for myogenic index in µpatterned tissues. All data was not normally distributed, as determined by the Lilliefors test. The *p*-value for the Kruskal-Wallis test was 0.01. Multiple comparisons were performed using Tukey's test, with *p*-values for statistical differences indicated in the table below.

Comparison	*p*-value
Wk1 FN-µprinted soft PDMS vs Wk1 µmolded gelatin	NS
Wk3 FN-µprinted soft PDMS vs Wk3 µmolded gelatin	<0.03
Wk1 FN-µprinted soft PDMS vs Wk3 µmolded gelatin	NS
Wk3 FN-µprinted soft PDMS vs Wk1 µmolded gelatin	<0.03
Wk1 FN-µprinted soft PDMS vs Wk3 FN-µprinted soft PDMS	NS
Wk1 µmolded gelatin vs Wk3 µmolded gelatin	NS

Supplementary Table S7. Statistical analysis for myogenic index in isotropic tissues. All data was not normally distributed, as determined by the Lilliefors test. The *p*-value for the Kruskal-Wallis test was 7.0 x 10e-4. Multiple comparisons were performed using Tukey's test, with *p*-values for statistical differences indicated in the table below.

Comparison	*p*-value
Wk1 FN-isotropic soft PDMS vs Wk1 isotropic gelatin	NS
Wk3 FN-isotropic soft PDMS vs Wk3 isotropic gelatin	<0.03
Wk1 FN-isotropic soft PDMS vs Wk3 isotropic gelatin	NS
Wk3 FN-isotropic soft PDMS vs Wk1 isotropic gelatin	<0.01
Wk1 FN-isotropic soft PDMS vs Wk3 FN-isotropic soft PDMS	NS
Wk1 isotropic gelatin vs Wk3 isotropic gelatin	NS

Supplementary Table S8. Statistical analysis for myotube width in µpatterned tissues. All data was not normally distributed, as determined by the Lilliefors test. The *p*-value for the Kruskal-Wallis test was 0.01. Multiple comparisons were performed using Tukey's test, with *p*-values for statistical differences indicated in the table below.

Comparison	*p*-value
Wk1 FN-µprinted soft PDMS vs Wk1 µmolded gelatin	NS
Wk3 FN-µprinted soft PDMS vs Wk3 µmolded gelatin	<0.01
Wk1 FN-µprinted soft PDMS vs Wk3 µmolded gelatin	NS
Wk3 FN-µprinted soft PDMS vs Wk1 µmolded gelatin	NS
Wk1 FN-µprinted soft PDMS vs Wk3 FN-µprinted soft PDMS	NS
Wk1 µmolded gelatin vs Wk3 µmolded gelatin	NS

Supplementary Table S9. Statistical analysis for myotube width in isotropic tissues. All data was not normally distributed, as determined by the Lilliefors test. The *p*-value for the Kruskal-Wallis test was 3.4 x 10e-4. Multiple comparisons were performed using Tukey's test, with *p*-values for statistical differences indicated in the table below.

Comparison	*p*-value
Wk1 FN-isotropic soft PDMS vs Wk1 isotropic gelatin	NS
Wk3 FN-isotropic soft PDMS vs Wk3 isotropic gelatin	<0.001
Wk1 FN-isotropic soft PDMS vs Wk3 isotropic gelatin	<0.02
Wk3 FN-isotropic soft PDMS vs Wk1 isotropic gelatin	<0.03
Wk1 FN-isotropic soft PDMS vs Wk3 FN-isotropic soft PDMS	NS
Wk1 isotropic gelatin vs Wk3 isotropic gelatin	NS

Supplementary Table S10. Statistical analysis for myotube length in µpatterned tissues. All data was not normally distributed, as determined by the Lilliefors test. The *p*-value for the Kruskal-Wallis test was 1.3 x 10e-5. Multiple comparisons were performed using Tukey's test, with *p*-values for statistical differences indicated in the table below.

Comparison	*p*-value
Wk1 FN-µprinted soft PDMS vs Wk1 µmolded gelatin	<0.05
Wk3 FN-µprinted soft PDMS vs Wk3 µmolded gelatin	<0.0001
Wk1 FN-µprinted soft PDMS vs Wk3 µmolded gelatin	<0.01
Wk3 FN-µprinted soft PDMS vs Wk1 µmolded gelatin	<0.001
Wk1 FN-µprinted soft PDMS vs Wk3 FN-µprinted soft PDMS	NS
Wk1 µmolded gelatin vs Wk3 µmolded gelatin	NS

Supplementary Table S11. Statistical analysis for myotube length in isotropic tissues. All data was not normally distributed, as determined by the Lilliefors test. The *p*-value for the Kruskal-Wallis test was 2.0 x 10e-5. Multiple comparisons were performed using Tukey's test, with *p*-values for statistical differences indicated in the table below.

Comparison	*p*-value
Wk1 FN-isotropic soft PDMS vs Wk1 isotropic gelatin	<0.01
Wk3 FN-isotropic soft PDMS vs Wk3 isotropic gelatin	<0.01
Wk1 FN-isotropic soft PDMS vs Wk3 isotropic gelatin	NS
Wk3 FN-isotropic soft PDMS vs Wk1 isotropic gelatin	<0.0001
Wk1 FN-isotropic soft PDMS vs Wk3 FN-isotropic soft PDMS	NS
Wk1 isotropic gelatin vs Wk3 isotropic gelatin	NS

Supplementary Table S12. Statistical analysis for myotube alignment on µmolded gelatin hydrogels. All data was normally distributed, as determined by the Lilliefors test. The *p*-value for one-way analysis of variance (ANOVA) was 9.3 x 10e-6. Multiple comparisons were performed using Tukey's test, with the *p*-values indicated in the table below.

**Comparison**	***p*-value**
Wk1 isotropic gelatin vs Wk3 isotropic gelatin	NS
Wk1 isotropic gelatin vs Wk1 µmolded gelatin	<0.001
Wk1 isotropic gelatin vs Wk3 µmolded gelatin	<0.01
Wk3 isotropic gelatin vs Wk1 µmolded gelatin	<0.001
Wk3 isotropic gelatin vs Wk3 µmolded gelatin	<0.01
Wk1 µmolded gelatin vs Wk3 µmolded gelatin	NS
